# Supplementary material for: Genome-Wide Identification and Expression Analysis of Growth-Regulating Factor Family in Sweet Potato and Its Two Relatives
Source: Genes (Basel). 2024 Aug 12;15(8):1064. doi: 10.3390/genes15081064 (PMC11353427; doi:10.3390/genes15081064)
Supplement: Supplementary file 1 [file genes-15-01064-s001.zip › Supplemental figure.pdf]

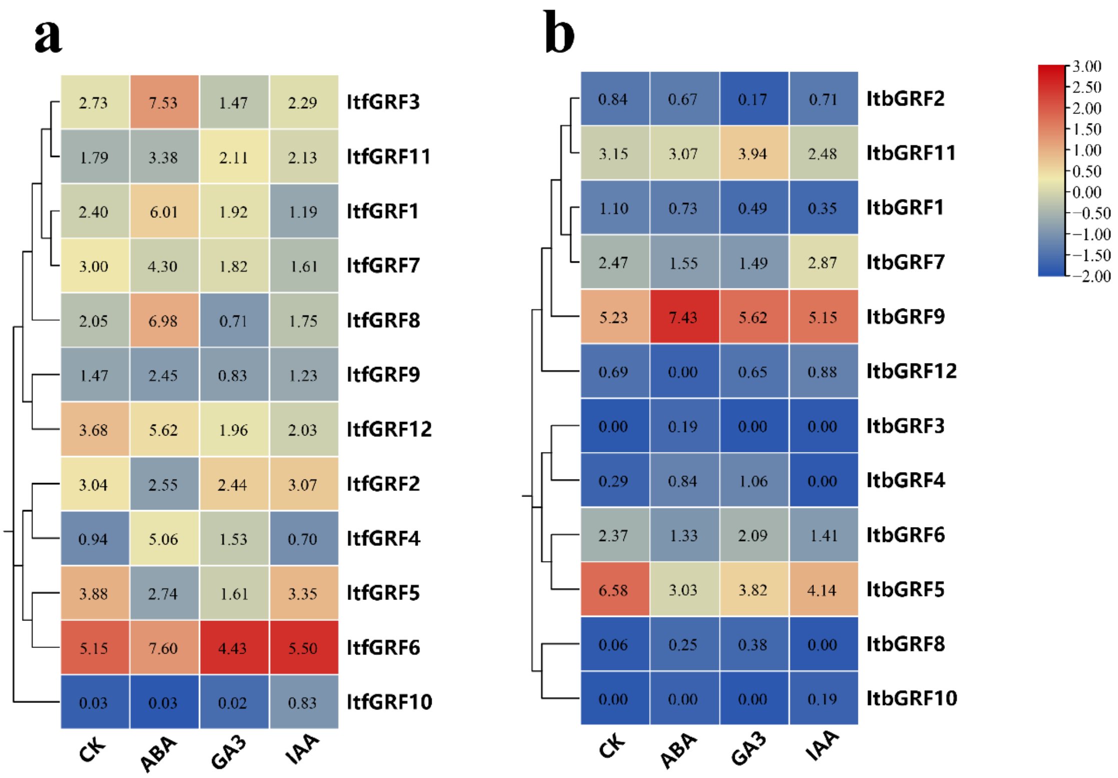

Figure S1. Expression analysis of *I.trifida* (**a**) and *I.triloba* (**b**) under hormones treatment (ABA, GA3 and IAA) as determined by RNA−seq. The figure value is the true expression, and the scale value is standardized according to the true expression, which reflects the relative expression value.

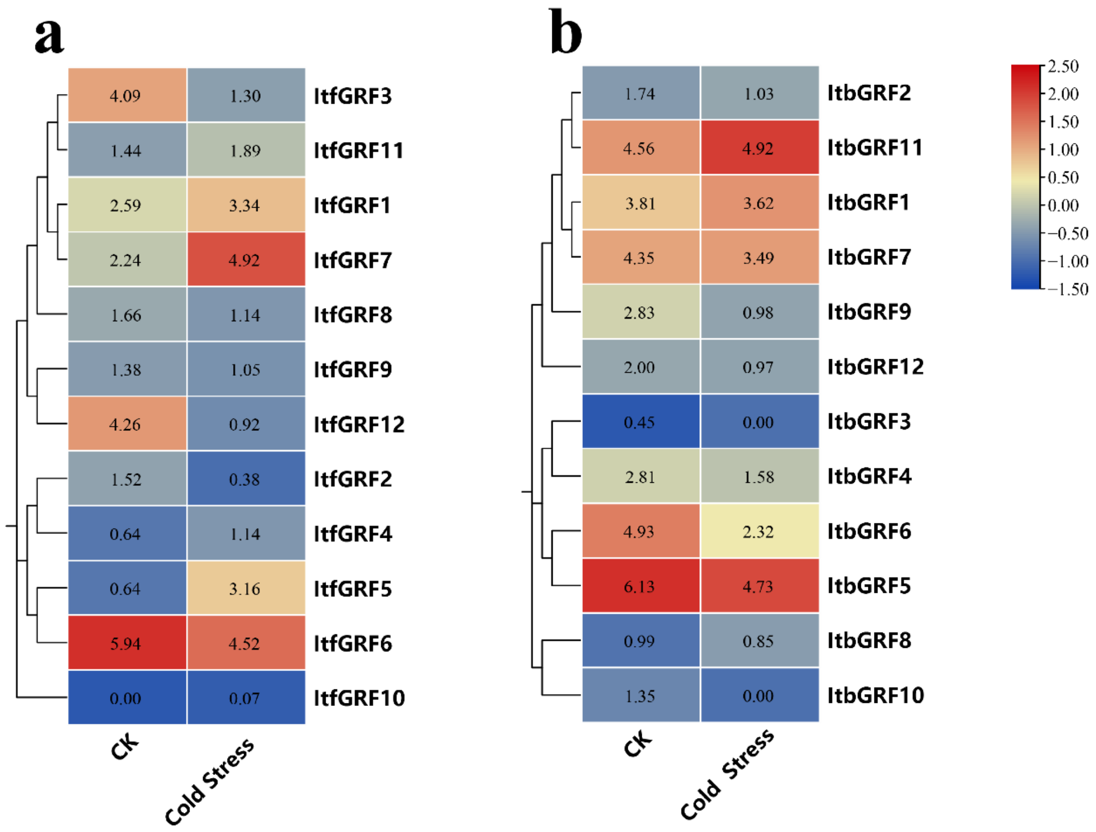

Figure S2. Gene expression patterns of *I.trifida* (**a**) and *I.triloba* (**b**) under cold stress as determined by RNA−seq. The figure value is the true expression, and the scale value is standardized according to the true expression, which reflects the relative expression value.

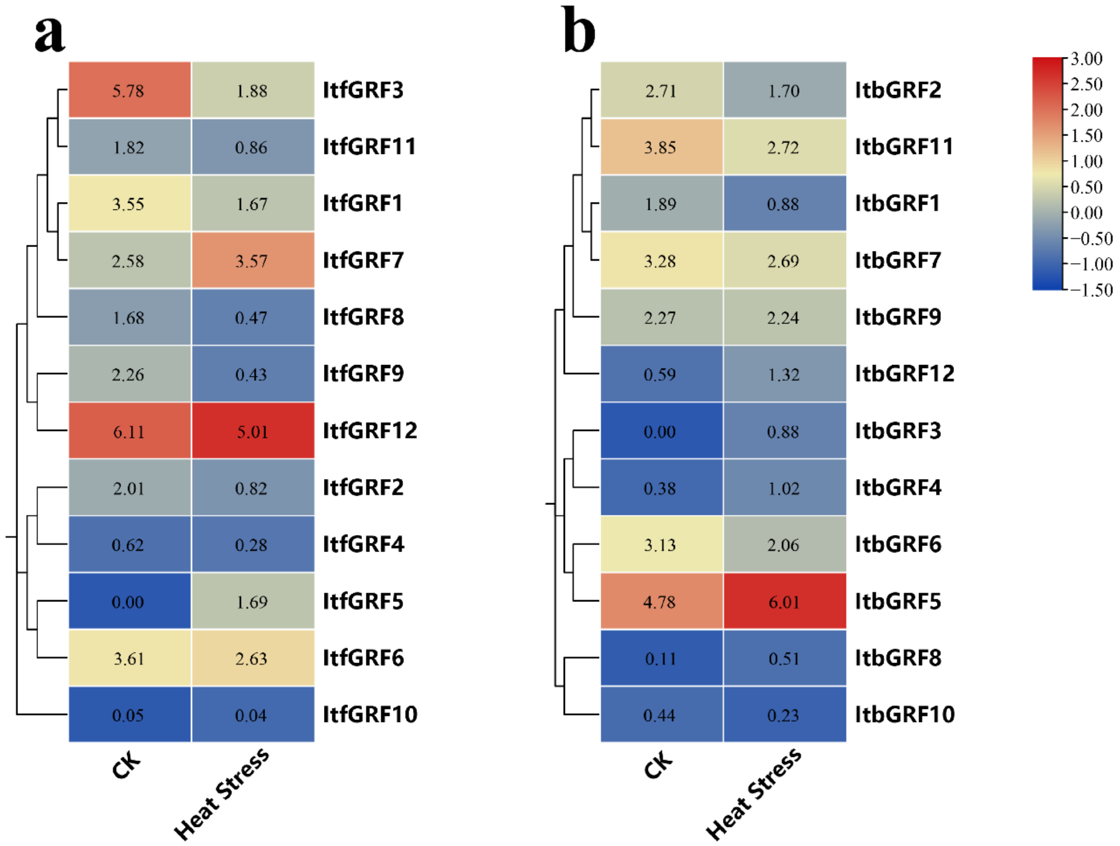

Figure S3. Gene expression patterns of *I.trifida* (**a**) and *I.triloba* (**b**) under heat stress as determined by RNA−seq. The figure value is the true expression, and the scale value is standardized according to the true expression, which reflects the relative expression value.

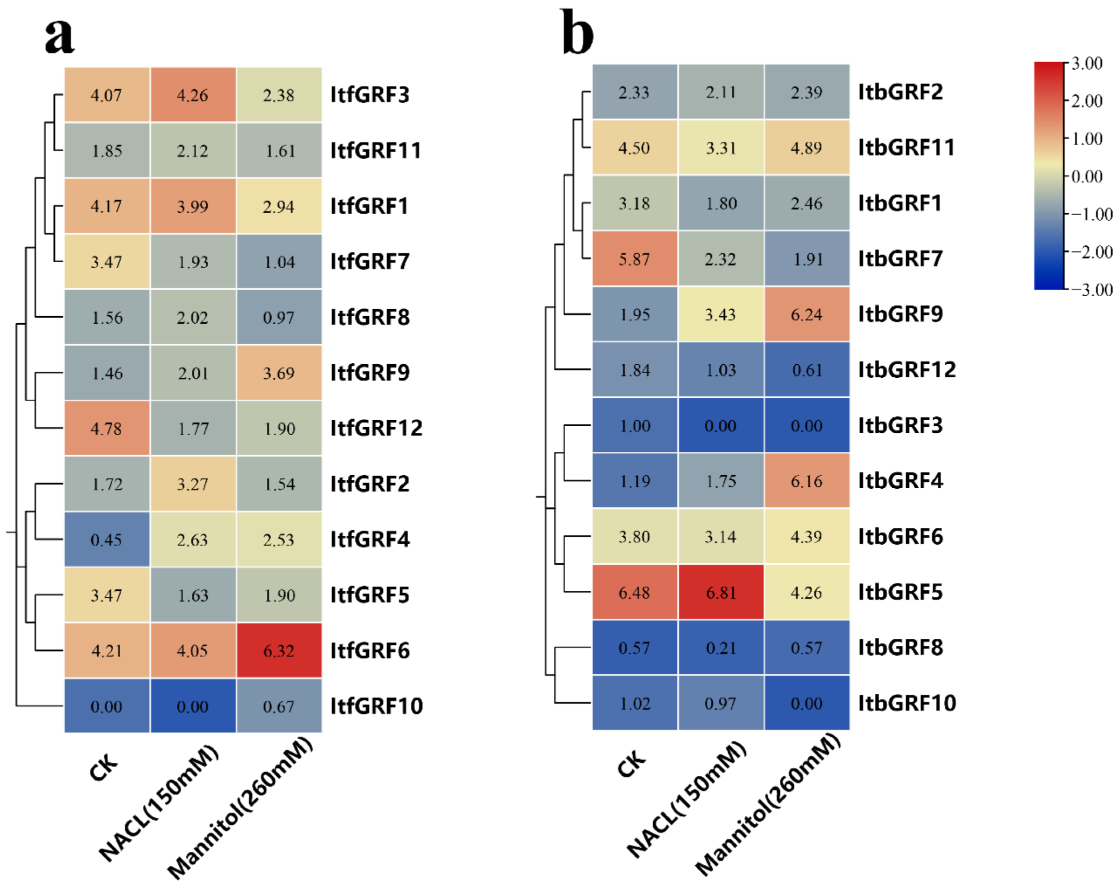

Figure S4. Gene expression patterns of *I.trifida* (**a**) and *I.triloba* (**b**) under salt and drought Stresses. NACL: salt stress; MANN: drought stress. The figure value is the true expression, and the scale value is standardized according to the true expression, which reflects the relative expression value.
